# On the Way to Experimental Test of the Time Reversal Invariance in the Nuclear Reactions

**DOI:** 10.6028/jres.110.073

**Published:** 2005-08-01

**Authors:** Vadim R. Skoy, Takashi Ino, Yasuhiro Masuda, Suguru Muto, Guinyun Kim

**Affiliations:** Frank Laboratory of Neutron Physics, Joint Institute for Nuclear Research, 141980 Dubna, Moscow Region, Russia; Institute of Materials Structure Science, High Energy Accelerator Research Organization, Tsukuba, Ibaraki, 305-0801, Japan; Institute of High Energy Physics, Kyungpook National University, 1370 Sankyok-dong, Buk-gu, Daegu 702-701, Korea

**Keywords:** polarized neutrons, polarized nuclei, time reversal invariance

## Abstract

Time (T) violation can be related with charge-parity (CP) violation through the CPT theorem. The CP violation was discovered experimentally in the K_0_-meson decays about 35 years ago. The T violating interaction related with the CP violation violates parity as well. However, an extension of the theory beyond the locality of the interactions might violate the CPT theorem. The result of the CPLEAR experiment [1], which has given direct evidence of T violation in the elementary-particle phenomena, could be considered under assumption of the CPT invariance.

## 1. T-Violating Neutron Transmission Experiment

In the K_0_-meson decay, CP-violating interactions belong to the Δ*S* = 1 sector. However, some models predict large CP-violation effects for the Δ*S* = 0 sector. From this point of view, a low energy phenomenon (Δ*S* = 0) is very interesting because it can give independent additional information about CP-violation. It means that searches for the simultaneous P and T violations in nonleptonic processes are still of great importance. The contribution of Kobayashi-Maskawa phase to the corresponding observables is very small. Thus, such experiments probe new types of T-violating interactions. At present, the study of polarized neutron transmission through polarized targets seems to be one of the most promising ways to test time reversal invariance. This is due to the dynamical and resonance enhancement factors which act in both cases of P-violating (P-odd) and T-violating (T-odd) interactions and causes the increase in the violation effects near neutron p-wave resonances.

The transmission experiments have principal advantage in comparison with any other kinds of the decay experiments. The behavior of this advantage originates exactly from the behavior of the time reversal as a symmetry operation itself. Namely, unlike the other symmetries (space, charge, etc.), not only does the time reversal alter the signs of some quantities, time reversal replaces initial and final states of any process as well. Hence, the transmission processes, where the initial and final states of reaction coincide by definition, match very well the requirements of the time reversal invariance check-up. The decay reactions, where the initial and final states are different, have limited abilities for this problem [2]. The principal limitation arises from final state interactions. For example, in the neutron *β*-decay experiments the limitation arises from electron-proton Coulomb scattering.

The above mentioned enhancement of the symmetry breaking effects in the neutron reactions with heavy nuclei originates from the multi-component of the nuclear compound states, namely, a big number of single particle nuclear configurations, which contribute to the excited nuclear state after compound nucleus been formed. The correspondent matrix element between the initial and final compound states can be expressed as

$$w_{p}\langle \psi_{f}^{-}|v_{p}|\psi_{i}^{+}\rangle ,$$ (1)

where *v_P_* is a single particle matrix element of the P-odd interaction, and $\begin{array}{c} \pm \\ i,f \end{array}$ are the eigenfunctions of the nuclear T-invariant Hamiltonian with the appropriate boundary conditions [2,3]. In the particular case of the bound states, these functions are commonly considered as states with opposite parities.

Further, if T-violating interaction exists, then the matrix element in Eq. (1) is not pure real (or imaginary) any more. In other words, its phase *λ* differs from 0 (or π):

$$v_{P}\to v_{P}e^{i\lambda }\approx w_{P}+iw_{P}\lambda =w_{P}+iw_{PT},$$ (2)

because within the frames of the all theoretical expectations, *λ* << 1. Here $w_{PT}$ is the matrix element of the T-violating interaction, and the phase is $\lambda =w_{PT}/w_{P}$. Table 1 shows the expected magnitude of the quantity *λ* from different theoretical models [3]. The variation of theoretical prediction is quite large. However, at least the half of the models are listed in the Table 1 may be tested experimentally on the forthcoming generation of neutron sources.

## 2. The General Features of P-Odd and T-Odd Effects in Neutron Transmission

The transmission of neutrons through a polarized target can be described by the forward scattering amplitude, which in the spinor’s space has a form,

f=A+B(s⋅I)+C(s⋅k)+D(s⋅[k×I]. (3)

Here, ***s*** is the neutron spin, ***I*** the spin of the target nuclei, and ***k*** the neutron wave vector. The coefficient *A* represents the effect of a neutron spin independent strong interaction. The coefficient *B* represents the combined effects of an external magnetic field and nuclear pseudomagnetism. The coefficient *C* is the amplitude of the weak P-odd interaction. The coefficient *D* represents the amplitude of the P-odd and T-odd interaction. The real parts of the spin dependent terms cause neutron-spin precessions about the directions of ***I***, ***k***, and [***k*** × ***I***]. The imaginary parts lead to the attenuations of neutron-spin states in these three vector directions.

**Fig. 1:**
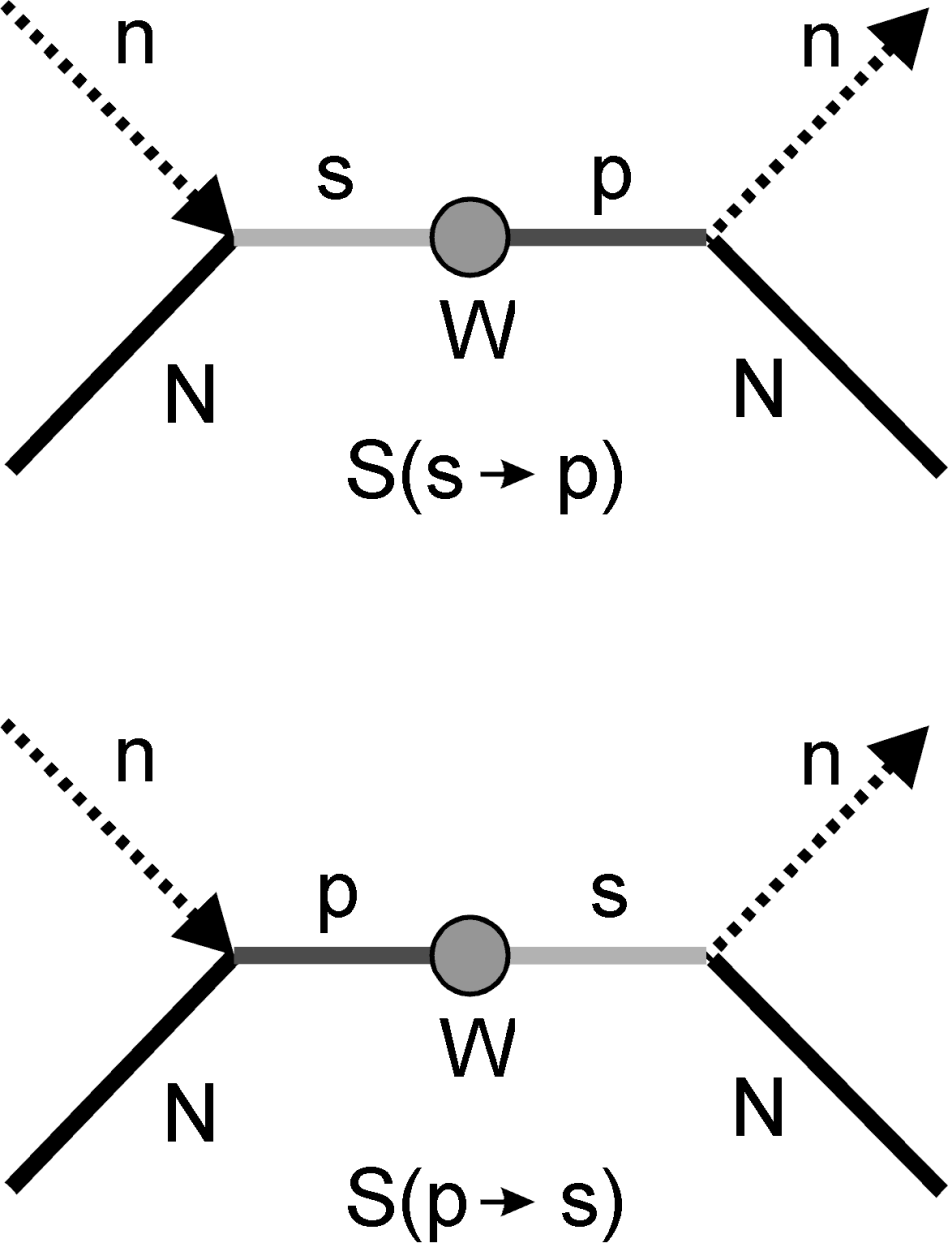
Diagrams of P-odd and T-odd interactions.

The terms *D* and *C* are quite similar in their structure, which arises from the two diagrams shown in Fig. 1. The left diagram represents a neutron capture by a target nucleus, which results in an s-wave compound resonance state (positive parity). Then, the weak interaction (circle) transforms this state to a p-wave state (negative parity). Finally, the compound nucleus decays with an outgoing neutron. The right part of Fig. 1 represents the similar process, but the final state is the s-wave state, which is transformed from the p-wave initial capture state. It is easy to see that the right picture is transformed to the left one under time reversal.

Thus, within the usual S-matrix approach [2,4],

$$\begin{array}{l} C\approx S(s\to p)+S(p\to s)\sim w_{P}\\ D\approx S(s\to p)-S(p\to s)\sim w_{PT}, \end{array}$$ (4)

where the *S* denotes the elements of the *S*-matrix. If the T-invariance holds, then there is no difference between the above two diagrams. Hence, if the term D ≠ 0, it means violation of T-invariance.

Now, let us assume the neutron beam direction is ***k*** along a *z* axis, the nuclear spin a *y* axis and then an *x* axis is given by [***k*** × ***I***]. In general case, a polarization vector in this frame has three components as ***p*** = (*p_x_*, *p_y_*, *p_z_*).

**Fig. 2:**
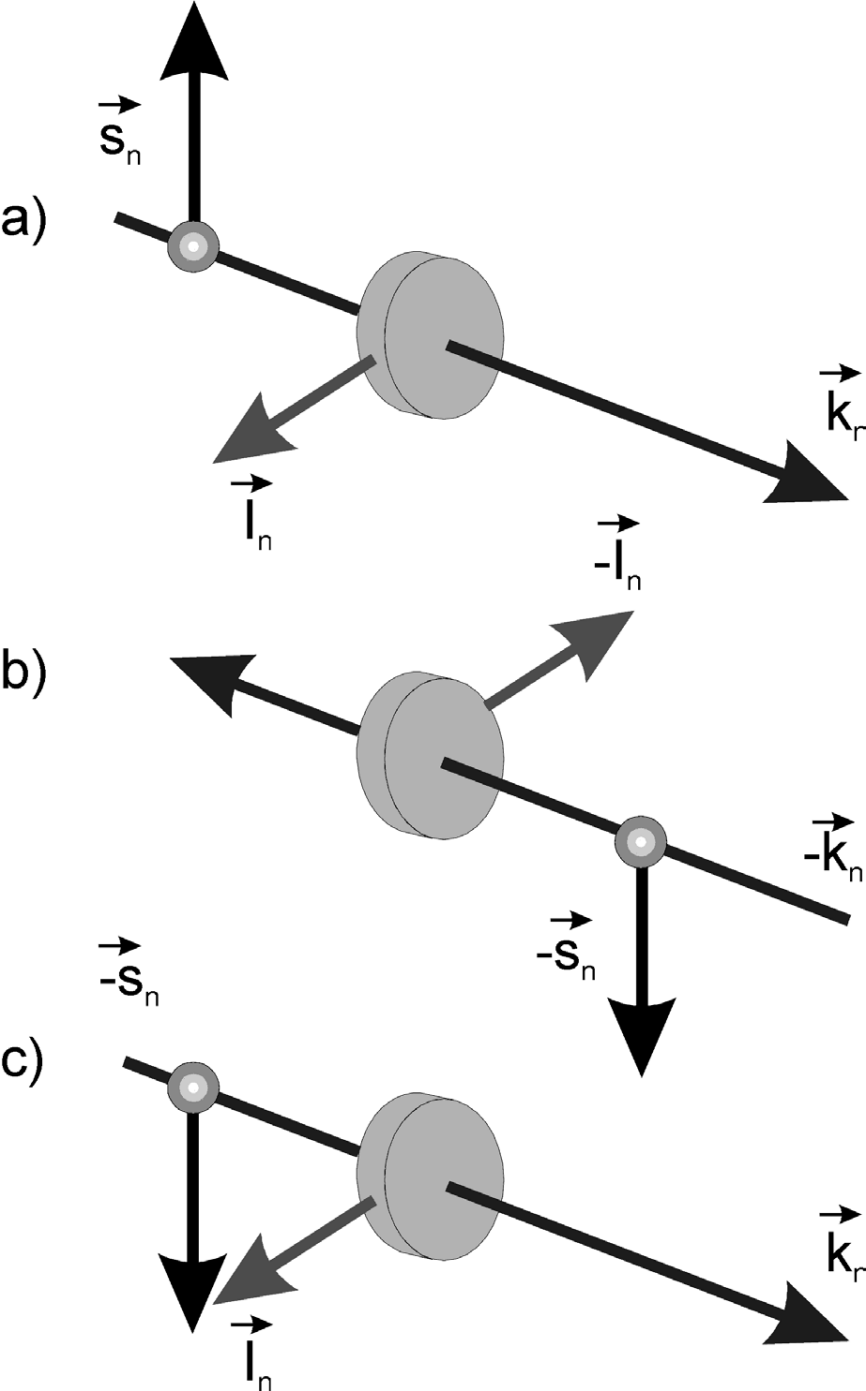
Effect of T-violation in neutron transmission.

At a first glance, the measurement of the term *D* is quite simple as it is shown in Fig. 2. Indeed, picture b) in Fig. 2 is just the time reversal pattern of picture a). The initial state is replaced with the final state. However, since we can not change the direction of the neutron beam, picture b) should be rotated by 180° around the *x* axis. Finally, we get picture c) which differs from a) by the neutron-spin direction only. Hence, to check the T-invariance, one should check the transmission difference when the neutron polarization is reversed.

However, in practice, the situation is more complicated owing to the presence of the strong interaction term *B*. Its real part makes the neutron spin rotate around the direction of the nuclear polarization. Even if the neutron polarization aligns exactly along the *x* axis at the entrance of target, its *z* component arises during the neutron beam propagation. As a result, the P-odd term C gives rise to the transmission difference, even if T-violating interaction absents at all. Hence, the experimental technique must be modified to exclude this kind of false effect.

## 3. Feasibility of the Experiment

**Fig. 3:**
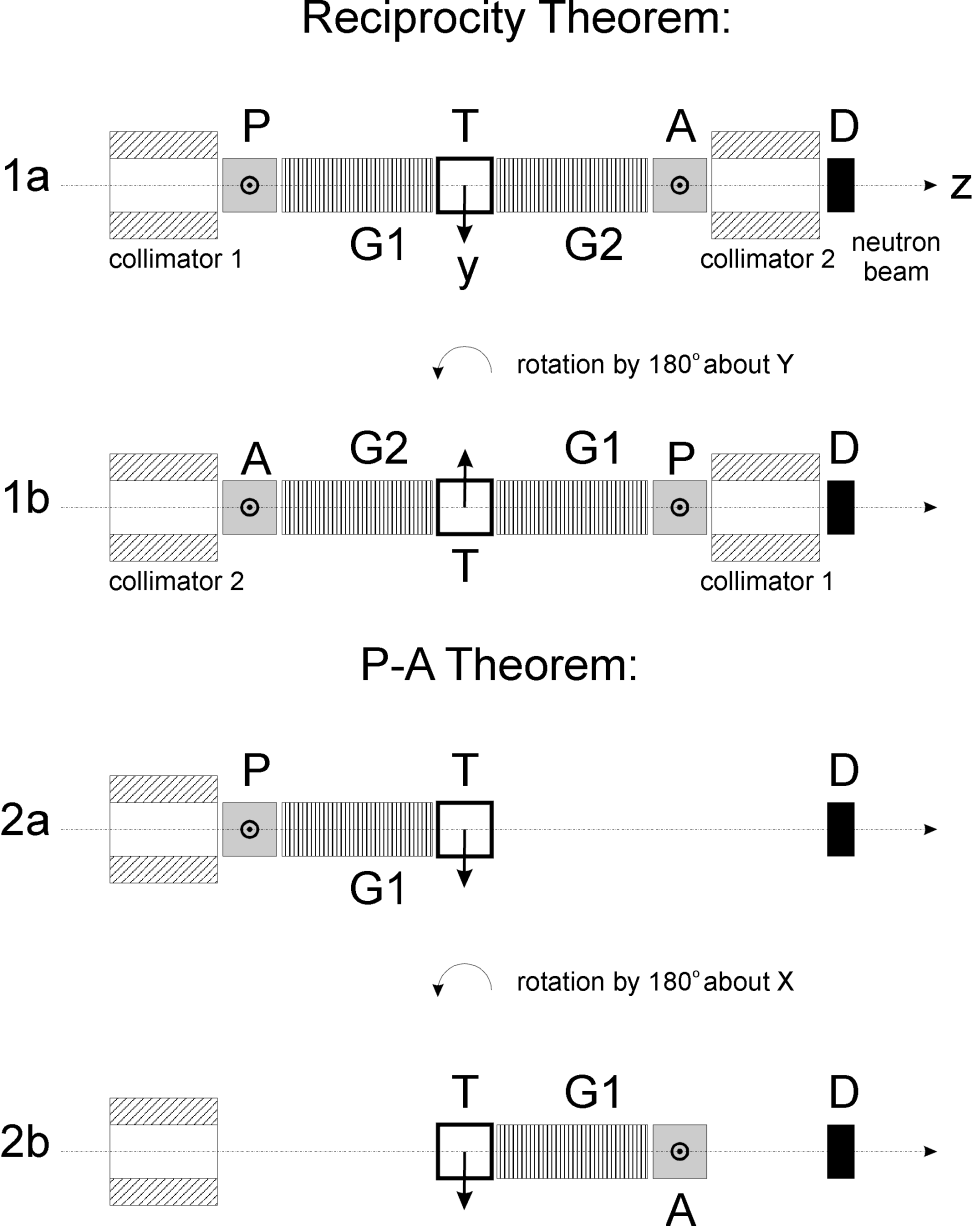
Two proposals for T-violation experiments.

We shall describe the two experimental methods which have been proposed in Ref. [5] and [6]. Both of them are shown in Fig. 3. The Ref. [5] discusses the application of a reciprocity theorem to the cancellation of any T-invariant false effect. Figure 3.1a and 3.1b describe one of the T-violation experiments. The neutron polarization is along the *x* axis which is perpendicular to the picture’s plane. We measure a neutron transmission *N_ap_* in the configuration shown in Fig. 3.1a. Neutrons first pass through the neutron spin polarizer (P), second the polarized target (T), third the neutron spin analyzer (A), and then are registered by the detector (D). We also measure a neutron transmission *N_ap_* in the configuration shown in Fig. 3.1b. We rotate the entire set of equipment including the target, neutron spin guide channels (G1 and G2) and collimators, about the *y* axis by 180°. The directions of the polarization axes of the polarizer and analyzer are reversed after rotation as well. The target polarization is also reversed. After these operations, Fig. 3.1a transforms to Fig. 3.1b. The transformation is a kind of complete time reversal for the experimental apparatus. The polarization components of the polarizer *p_x,y,z_* and analyzer, *a_x,y,z_* transform in the following way.

$$\begin{array}{l} \left(p_{x},p_{y},p_{z})\to (p_{x},-p_{y},p_{z}\right)\\ (a_{x},a_{y},a_{z})\to (a_{x},-a_{y},a_{z}), \end{array}$$ (5)

The difference between the two transmissions is

Npa−Nap~(px+ax)Im(D′)+(aypz−azpy)Re(D′)++(pz−az)Im(B′D′)+(axpy+aypx)Re(B′D′)++(py−ay)Im(C′D′)+(axpz+azpx)Re(C′D′). (6)

Here, *B*′ = *B*sin(*qt*)/*q*, *C*′ = *C*sin(*qt*)/*q*, *D*′ = *D*sin(*qt*)/*q*, *t* is the time of flight through the target, and q ∼ Re(*B*) + *i*Im(*B*).

All the terms in Eq. (6) are proportional to the desired term *D* as a result of the reciprocity theorem. The false effects due to polarizer and analyzer misalignments [7] as well as the effect of magnetic field leakage from the target region vanish. From expression for *D*′, it follows that one must suppress the term Re(*B*) >> Im(*B*), because it comes to the denominator *q*. This behavior of the *B* term was first pointed out in Ref. [8]. The solution for this problem comes from the common features of the pseudomagnetic and magnetic fields. Both of them make the neutron spin rotate around their directions. It means that Re(*B*) can be considered as an effective field *H*_0_. Hence, one may apply an opposite external field to compensate the effect of *H*_0_.

Figures 3.2a and 3.2b show the test of T-invariance by using the polarization-asymmetry (P-A) theorem [6]. If T-invariance holds, then an asymmetry in polarized neutron transmission must be exactly equal to the neutron polarization, which is initially zero, produced upon transmission. The asymmetry is obtained from a difference, Δ*N_p_*, in the transmissions through the target T for the two opposite neutron polarization states, which are produced by the polarizer P (Fig. 3.2a). For the polarization measurement the polarizer P is rotated around the target by 180° (Fig. 3.2b). The rotation axis coincides with the *x* axis. Thus, the polarizer P in Fig. 3.2a becomes the analyzer A in Fig. 3.2b after the rotation. However, the target remains in its initial position and its polarization is not changed. After the rotation, the components of the polarization vector are transformed in the obvious way,

$$\left(p_{x},p_{y},p_{z})\to (p_{x},-p_{y},-p_{z}\right)$$ (7)

If we reverse the polarization of the analyzer, then we obtain a difference Δ*N_a_*, which arises from the neutron polarization upon transmission through the target. Thus we obtain two kinds of differences,

ΔNp~px[Im(D′)+Im(B′C′)]+py[Im(B′)+Im(C′D′)]+pz[Im(C′)+Im(B′D′)]ΔNa~px[Im(D′)−Im(B′C′)]−py[Im(B′)−Im(C′D′)]−pz[Im(C′)−Im(B′D′)] (8)

The sum of these quantities gives

ΔNp+ΔNa~pxIm(D′)+pyIm(C′D′)+pzIm(B′D′) (9)

All the terms are proportional to *D*. The terms independent from *D* are excluded due to the transformation properties of the neutron’s polarization vector. In this method the difference between polarizer and analyzer is absent completely, because they are the same device. The misalignments of the polarizer axis may just decrease the observed true effect, but at least do not provide false effects.

## 4. Expected T-Violation Effect

We have two possibilities for polarized target nuclei, ^139^La and ^131^Xe. ^139^La can be polarized in a single crystal of LaAlO_3_ by a dynamical nuclear polarization [9,10] and ^131^Xe by an optical pumping [11].

The pseudomagnetic precession must be compensated with an external magnetic field in order to avoid the suppression of the true T-violation effect. For ^131^Xe, the pseudomagnetism is compensated with other noble gases (for example ^129^Xe, ^83^Kr, ^21^Ne) polarized by the same optical pumping, if some of these nuclei has the opposite sign of pseudomagnetic field in the vicinity of the p-wave resonance.

**Fig. 4:**
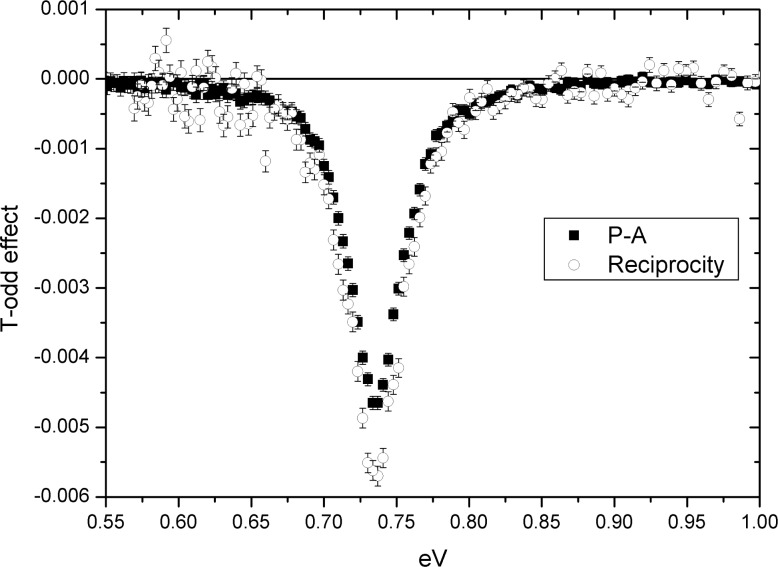
Monte Carlo simulations of T-odd effect.

We simulated the reciprocity and P-A theorem experiments for a LaAlO_3_ target by means of Monte Carlo calculation. In this simulation, we assumed *w_PT_* = *w_P_*. The results of the simulation for T-odd effects ∼ Im(*D*′) are shown in Fig. 4 as a function of the neutron energy near p-wave resonance 0.734 eV.

## 5. Polarized Lanthanum Target

We will polarize lanthanum nuclear spins by using a dynamic nuclear polarization upon a paramagnetic electron spin resonance (ESR) in a single crystal of a lanthanum compound. The dynamic polarization is well established by our group at KEK. Paramagnetic electrons are almost 100 % polarized in a strong magnetic field at low temperature. The electron spin polarization is transferred to nuclear spins upon ESR in matter, since the electron spins couple with the nuclear spins via hyperfine interactions. The electron spins flip upon photon irradiation with a frequency matching with a energy difference in electron magnetic states. The nuclear spins also flip upon the ESR via hyperfine interactions. The electron spin relaxation time is much shorter than the nuclear spin relaxation time. The electron spins quickly return to their original direction so that they flip again under the photon irradiation, while the nuclear spins remain flipped.

**Fig. 5:**
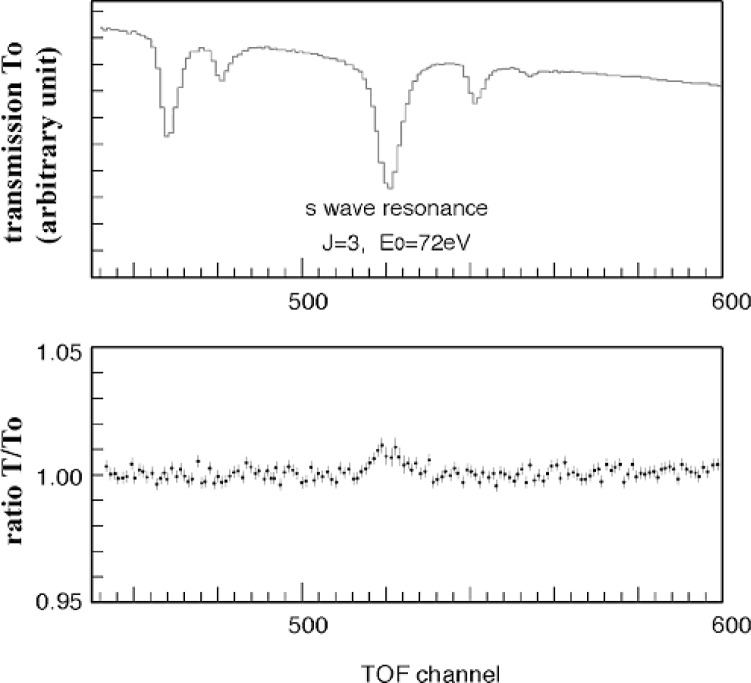
Neutron transmission of polarized LaAlO_3_.

We will use a single crystal of LaAlO_3_ for lanthanum nuclear polarization. We have carried out a proto-type experiment on the dynamic nuclear polarization on lanthanum nuclei [9,10]. We replaced 0.03 % of lanthanum atoms with neodymium atoms which work as paramagnetic centers. We placed the single crystal in a microwave cavity, which was located in a magnetic field of 2.5 T at a temperature of 0.5 K. The nuclear polarization was observed by a NMR. Besides, we measured a neutron transmission for the polarized LaAlO_3_ single crystal. The result is shown in Fig. 5. ^139^La has an s-wave resonance at 72 eV. Its total cross section for the polarized nucleus is represented as

$$\sigma_{\pm }=\sigma_{0}(1\pm \rho P_{I})$$ (10)

The signs depend on the neutron-nucleus spin states, which are ± for parallel and ant-parallel spin states, respectively, and *ρ* = −1 is a spin factor. The neutron transmittance of the polarized lanthanum nuclear target, *T* is represented in terms of a transmittance of unpolarized target *T*_0_, a nuclear polarization *P_I_*, *σ*_0_, a nuclear number density *n*, a target thickness *L*.

$$T=T_{0}\;\cosh(P_{I}\sigma_{0}nL)$$ (11)

The nuclear polarization of ^139^La was obtained to be 10 %–20 % from the measurement of *T* and *T*_0_ [9].

## 6. Polarized ^3^He and Xenon Targets

Nuclear polarizations for odd isotopes in any noble gases by means of optical pumping are quite similar. First, circularly polarized laser light polarizes rubidium atoms in the D1 resonance at a wavelength of 795 nm. Second, polarization of the rubidium atoms is transferred to the noble gas nuclei via hyperfine interactions during atomic collisions. The maximal nuclear polarization that can be achieved is

$$p_{I}^{\max}=P_{\text{Rb}}\frac{\gamma_{\text{SE}}}{\gamma_{\text{SE}}+\gamma },$$ (12)

where *P*_Rb_ is the rubidium atomic polarization, *γ* a ^3^He or xenon polarization relaxation rate, *γ*_SE_ a spin exchange rate between rubidium atoms and noble gas nuclei [12]. The xenon isotopes are polarized much faster than ^3^He nuclei, however, their relaxation rates are larger. Nuclei with a spin *I* = 1/2 lose their polarization by dipole-dipole interactions during collisions with walls or with each other, while nuclei with a spin *I* > 1/2 have an additional loss by electric quadrupole interactions. ^131^Xe nuclei are used as a polarized target, while the other noble gas nuclei with *I* ≠ 0 may be used for the compensation of the pseudomagnetism.

Neutron-^3^He interactions at low energies are dominated by the ^3^He(n,p)^3^H reaction which depends on a neutron-^3^He spin state. Because of the polarization cross section, neutrons are polarized after the transmission through polarized nuclei. The neutron polarization is expressed in the following equation.

$$p_{n}=\sqrt{1-{\left(T_{0}/T\right)}^{2}.}$$ (13)

**Fig. 6:**
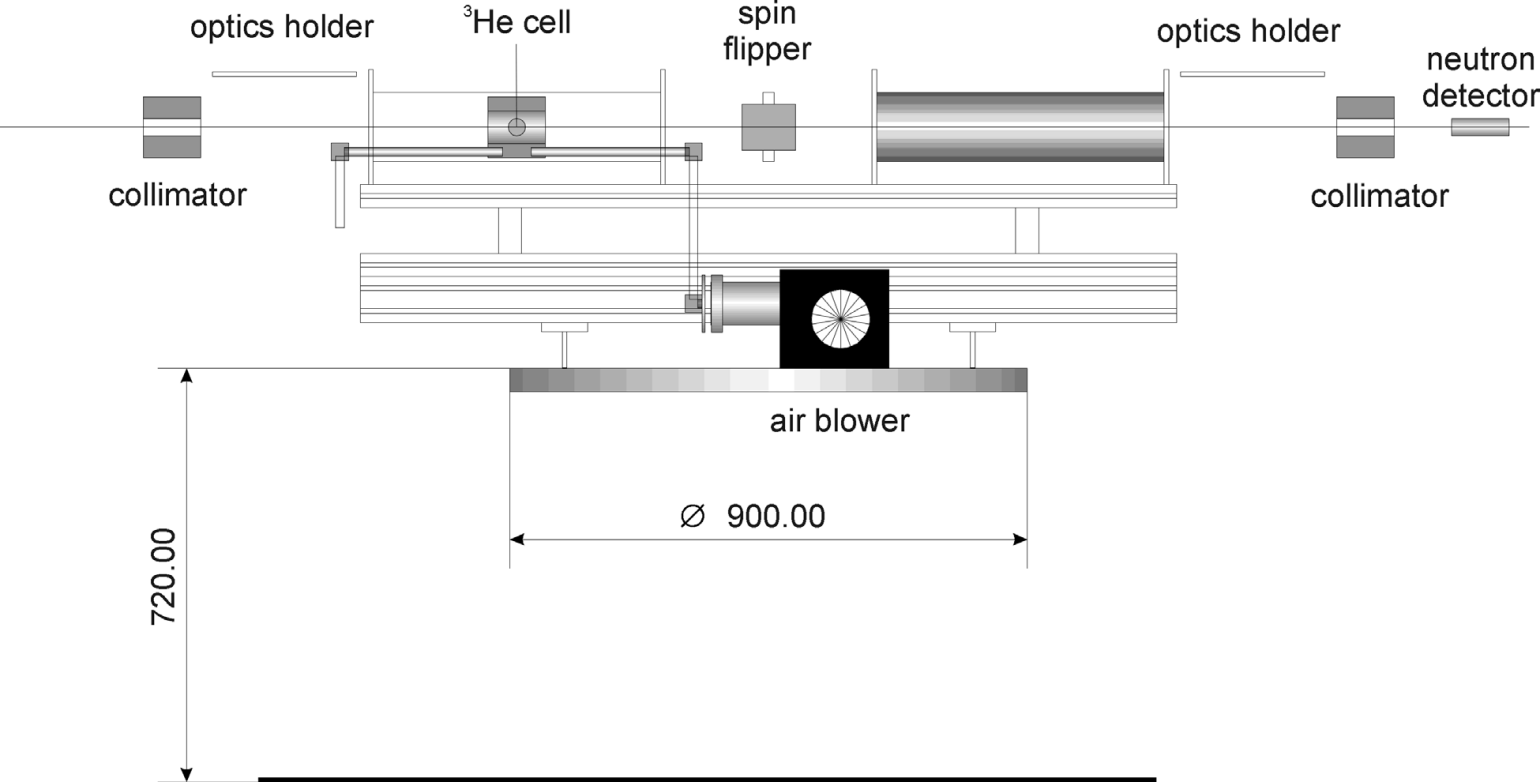
Polarized ^3^He neutron spin polarizer and analyzer in solenoids.

**Fig. 7:**
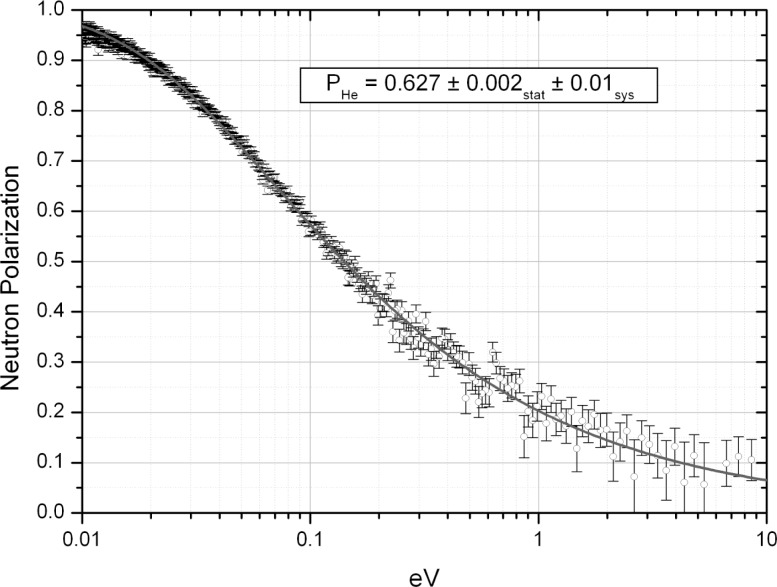
Neutron polarization by a 50 mm long at 3 bar ^3^He cell.

We are developing the ^3^He polarization to meet the above-mentioned requirements. We have successfully developed sapphire cylindrical helium cells with flat windows that can sustain a pressure of 3 bar. We built a proto-type of neutron polarizer and analyzer system with solenoids, which is shown in Fig. 6. Two ^3^He polarizers were placed on a rotating table in a KENS pulsed neutron beam line. We measured ^3^He polarizations and neutron polarizations by neutron transmissions. A typical result is shown in Fig. 7. Polarization of 62 % has been achieved. The length of the cell was 50 mm, and the wall thickness was 3 mm.

**Fig. 8:**
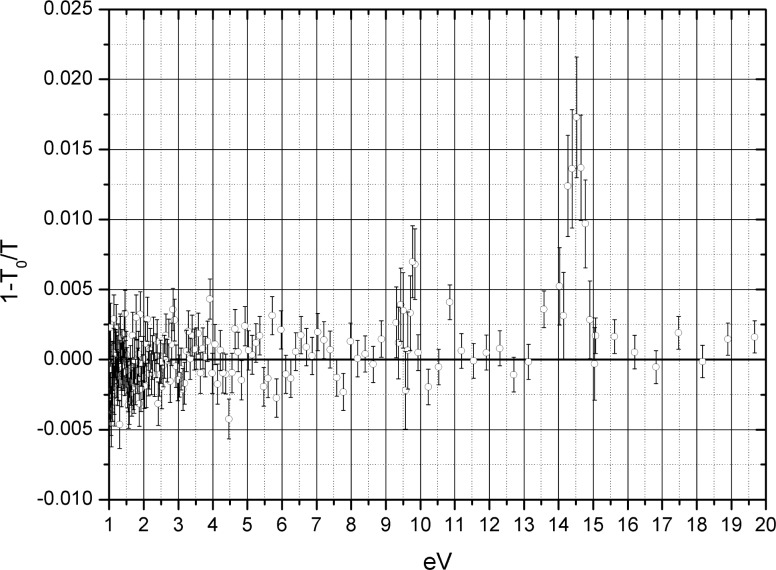
Neutron transmission enhancement by a 50 mm long at 1.3 bar natural xenon cell.

We also performed the optical polarization of natural xenon, which has two components with non zero spins ^129^Xe (*I* = 1/2) and ^131^Xe (*I* = 3/2). The procedure has been the same as for ^3^He. The quartz cell of 5 cm in length and with 1.3 bar of natural xenon was placed instead of helium cell (see Fig. 6). The extraction of the neutron polarization after transmission through the cell was done with Eq. (13). A preliminary result is shown in Fig. 8. We see the neutron transmission enhancement in vicinity of s-wave resonances 9.5 eV (^129^Xe) and 14.4 eV (^131^Xe). The ^131^Xe nuclear polarization *P_I_* was extracted from the Eq. (13) and Eq. (10) with spin factor *ρ* = 3/5 and found to be 20 %.

## Figures and Tables

**Table 1:** Theoretical predictions for the phase *λ*

Model	*λ*
Kobayashi – Maskawa	≤ 10^−10^
Right – Left	≤ 4 × 10^−3^
Horizontal Symmetry	≤ 10^−5^
Weinberg (charged Higgs bosons)	≤ 2 × 10^−6^
Weinberg (neutral Higgs bosons)	≤ 3 × 10^−4^
*θ*-term in QCD Lagrangian	≤ 5 × 10^−5^
Neutron EDM (one π-loop mechanism)	≤ 4 × 10^−3^
Atomic EDM (^199^Hg)	≤ 2 × 10^−3^
